# Automatic assessment of dairy cows' rumen function over time and links to feed changes and milk production

**DOI:** 10.3168/jdsc.2021-0165

**Published:** 2022-02-10

**Authors:** X. Song, S. van Mourik, E.A.M. Bokkers, P.W.G. Groot Koerkamp, P.P.J. van der Tol

**Affiliations:** 1 Farm Technology Group, Wageningen University and Research, PO Box 16, Wageningen, 6700 AA, the Netherlands; 2 Smart Component Department, Lely Industries N.V., Cornelis van der Lelylaan 1, Maassluis, 3147 PB, the Netherlands; 3 Animal Production Systems Group, Wageningen University and Research, PO Box 338, Wageningen, 6700 AH, the Netherlands

## Abstract

A 3-dimensional (3D) vision-based system was previously designed to automatically estimate the rumen motility of individual cows. This longitudinal study aimed to explore the associations between 3D vision-based rumen function assessment and dairy cow feed changes and milk production on a commercial farm. The 3D vision system was attached to an automatic milking robot to estimate the ruminal contraction frequency and rumen fill in 42 lactating cows during each milking event for 66 d. Additionally, we collected data on milk production, milk composition, general health, and changes in feeding practices. The 3D vision system showed that half the cows displayed a drastic decrease in the estimated rumen fill when all cows began grazing. The grazing and decreased rumen fill were also associated with herd-level milk fat depression. Over the 66 d, one cow was detected with reduced milk production and suspected rumen dysfunction by the farmer. The 3D vision system, however, identified this cow as having sudden decreases in estimated ruminal contraction frequency and rumen fill 4 d before detection by the farmer. In this longitudinal study, the 3D vision-based rumen function assessment system showed potential as a useful management-supporting tool for dairy farmers. The system, however, requires further validation with more cows of various breeds and ages. We suggest validating the 3D vision system with rumen boluses, quantified adjustments in feeding practices, more cases with ruminal dysfunction, and systematic health assessments for future studies.

## Highlights

• We monitored rumen function longitudinally in dairy cows using a 3-dimensional (3D) vision-based system.
• Rumen function was associated with feed changes and milk production.
• Early-spring grazing reduced rumen fill and milk fat content in cows.
• A cow with rumen dysfunction showed deviations in rumen motility in the 3D vision system.

## Graphical Abstract

**Figure:**
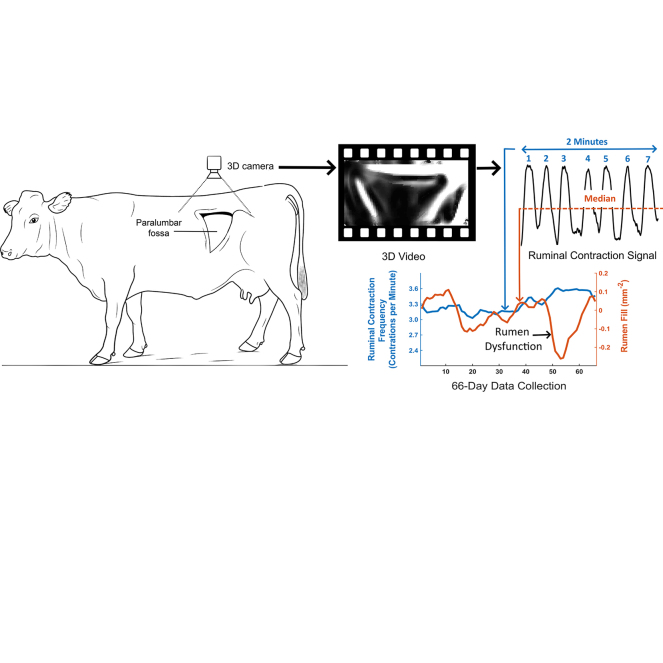
**Summary:** This study explored the associations between a 3-dimensional (3D) vision-based rumen function assessment and dairy cow feed changes and milk production. The 3D vision system showed that half the cows had a lowered rumen fill when they began grazing. Moreover, the system identified a cow with an empty and slowcontracting rumen before the farmer detected it. The 3D vision system showed potential for supporting farm management but requires further validation.

Rumen function in dairy cows is essential to digestive efficiency, milk production, and consequently farm profitability (Gross and Bruckmaier, 2019). It is commonly assessed by examining rumen motility, which is controlled by the parasympathetic nervous system. Examination of rumen motility is often performed by veterinarians on farms; however, it is labor intensive for veterinarians, costly for farmers, and consequently often requested by farmers only for cows suspected of being sick (Nagy, 2017). Sensor-based radiotelemetric rumen bolus systems have been developed to enable farmers to automatically and continuously measure the rumen temperature, pH, and motility of individual cows (Hamilton et al., 2019). These boluses provide direct information on the condition and function of the reticulum and rumen; however, they are costly, require insertion into individual cows, and the bolus battery life is short. Therefore, bolus systems are still not a feasible daily use solution for farmers. A low-cost, noninvasive, and long-lasting system is needed in order to automatically and regularly assess rumen function in dairy cows.

Recently, a low-cost 3-dimensional (**3D**) vision system was designed to automatically assess rumen motility (Song et al., 2019). This system remotely measures the morphological changes of the left paralumbar fossa of individual cows to identify ruminal contractions. This 3D vision system was verified by manual ruminal contraction detection and reached a matching sensitivity of 0.97. Although the 3D vision system showed promising results in identifying ruminal contractions and assessing rumen motility in a cross-sectional study, the feasibility of monitoring longitudinal changes in rumen motility of individual cows has not been tested. Additionally, this system should be a potential farm management tool to help farmers detect problems in dairy cow health, production, and feeding by monitoring rumen motility and function. The objective of the present research was to explore associations between the 3D vision-based rumen function assessment and dairy cow feed changes and milk production. This small-scale study was expected to demonstrate the potential of the 3D vision system in farm management and consequently to help set up direction and scope for future research.

The observational study was carried out on a Dutch commercial dairy farm and lasted for 66 d from March 8 to May 12, 2019. The observation included 42 Holstein Friesian × Brown Swiss crossbred lactating cows with an average parity of 1.2 (range 1–3). On d 66, the average DIM was 232 d (range 73–425 d). All cows were milked using an automatic milking system (**AMS**, Astronaut A5, Lely Industries N.V.) and fed indoors with an automatic feeder (Vector, Lely Industries N.V.).

The farmer managed common feeding at the herd level independent of this study. Every day, the farmer supplied 5.1 kg of concentrate supplements and 40 kg of fresh feed, containing 18 kg of DM per cow indoors. The feed consisted of grass silage, corn silage, barley, and hay with a composition ratio of 7:5:3:3 on a DM basis. From d 10 to 66, the farmer allowed all cows to graze on ryegrass-based pasture from 0600 to 1400 h each day, retaining the exact feed amounts and composition. From d 25 to 66, the farmer replaced hay with the same amount of corn silage.

A noninvasive general health check was performed by a veterinarian in training twice a week on all cows to detect health abnormalities. The veterinarian followed the protocol described by van Dixhoorn et al. (2018), including visual inspections of awareness, hair coat condition, breathing, and nose discharge, in addition to palpations for ear temperature (warm or cold) and udder condition. Thereafter, the veterinarian gave an overall health score for each cow by summing the number of examination aspects without any abnormality. The maximum score was 6, denoting healthy, and the minimum score was 0, denoting a great likelihood of having a health disorder. The general health checks were performed independently, and the results were not shared with the farmer.

The 3D vision system was designed to measure morphological changes of the left paralumbar fossa and estimate ruminal contraction frequency and rumen fill during each milking event, according to Song et al. (2019). The system contained a 3D camera (Realsense D415, Intel) that was mounted on the left-side fence of the AMS. The 3D camera was activated by digital signals from the AMS to record 3D video for approximately 4 min. The recording speed was reduced from 30 frames per second (**fps**), as used in the study of Song et al. (2019), to 4 fps in this study to minimize the video storage size and ensure sufficient memory space for long-term recording. A pilot study on video recording and image processing showed that a recording speed of 4 fps could still supply sufficient recording frames (i.e., approximately 40 frames per ruminal contraction) to identify ruminal contraction peaks and cycles. All video frames were analyzed to estimate the surface convexity of the left paralumbar fossa and chronologically assembled to form an oscillation signal. Then, the oscillation signal was processed by a discrete fast Fourier transform to determine the dominant frequency as the ruminal contraction frequency (min^−1^) and the average surface convexity of the left paralumbar fossa (mm^−2^) as the rumen fill. Both the estimated ruminal contraction frequency and rumen fill were saved after each successful milking of the 42 cows. Additionally, data from the farm management software (T4C, Lely Industries N.V.) in the AMS was collected, including estimated milk yield (kg), milk fat content (%), milk protein content (%), and milk fat-to-protein ratio. The distributions of the milk sensor data and the outputs of the 3D vision system for daily averages of all cows are presented in a group of boxplots (Figure 1). Additionally, the skewness and kurtosis of the distribution of each collected herd variable on each day were calculated (Figure 2) in MATLAB (R2018b, MathWorks) to describe the symmetry and outliers of the daily distribution.

**Figure 1:**
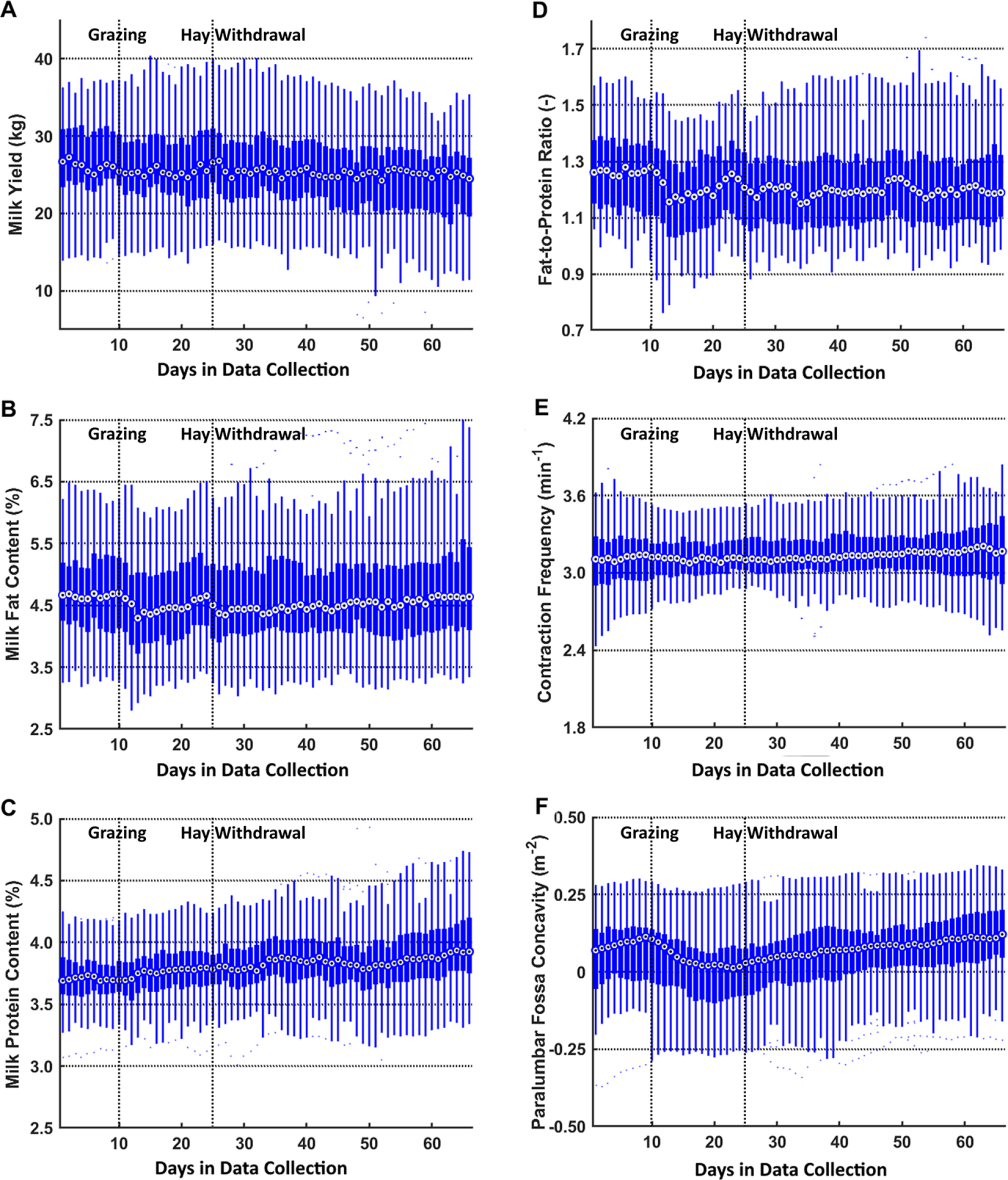
Boxplots of the outputs from the farm management software (A to D; T4C, Lely Industries N.V.) and 3-dimensional (3D) vision-based rumen motility assessment system (E and F; Song et al., 2019) of the herd over 66 d from March 8 to May 12, 2019. The output data include (A) milk yield (kg), (B) milk fat content (%), (C) milk protein content (%), (D) milk fat-to-protein ratio, (E) estimated ruminal contraction frequency (min^−1^), and (F) the surface concavity of the left paralumbar fossa as the estimated rumen fill (mm^−2^). In each box, the central mark is the median of the output signals of all cows on 1 d. The bottom and top edges of each box are the 25th (Q1) and 75th (Q3) percentiles of the output, respectively. The whiskers extend to the extreme data points that are within the range of [Q1 – 1.5 × (Q3 – Q1), Q3 + 1.5 × (Q3 – Q1)] and do not consider outliers. The outliers are located outside the range and are plotted individually as dots. The farmer allowed all cows to graze on d 10 and replaced hay with corn silage from indoor feeding on d 25.

**Figure 2:**
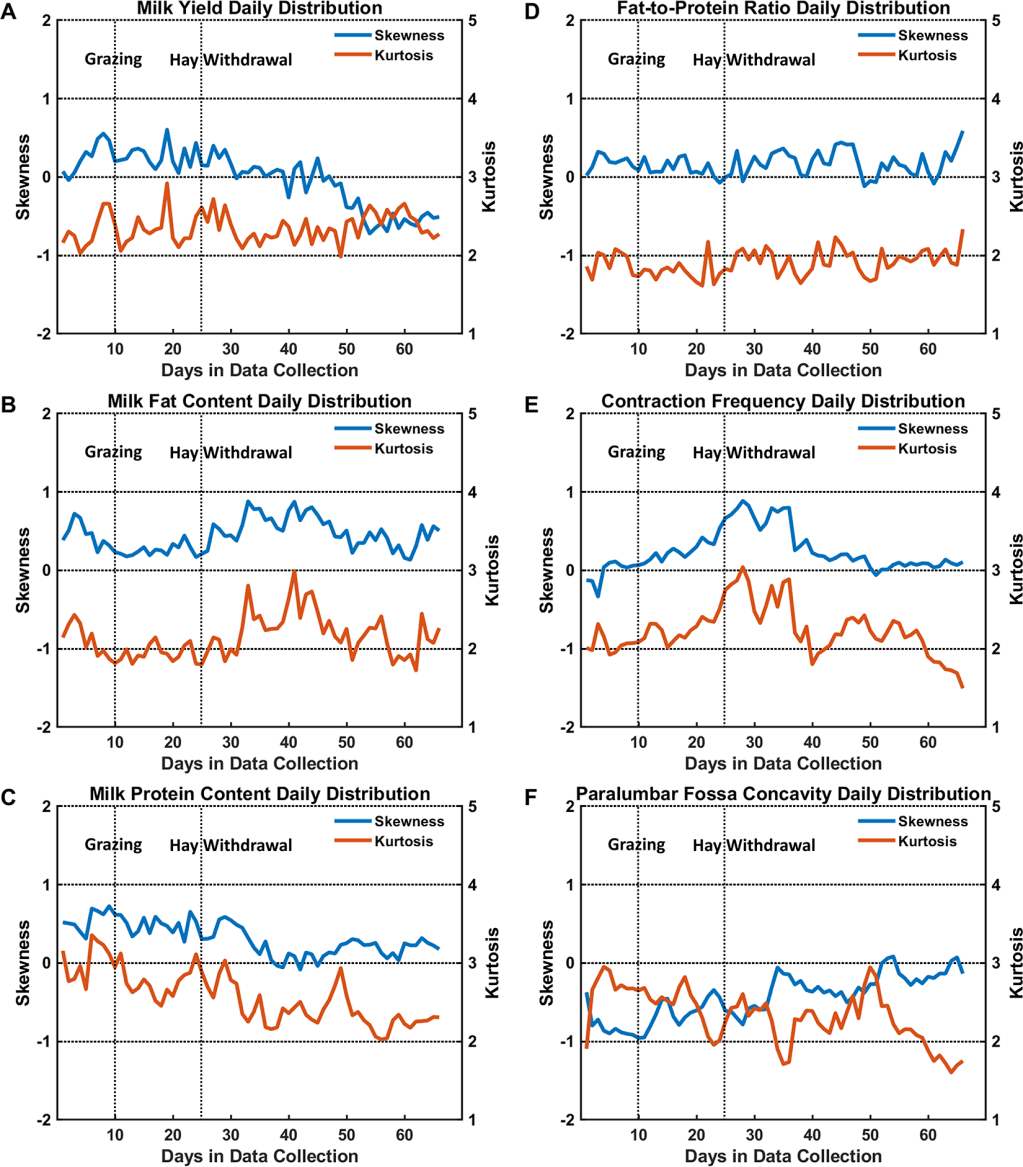
Plots of the skewness and kurtosis of the daily distribution from the farm management software (A to D; T4C, Lely Industries N.V.) and 3-dimensional (3D) vision-based rumen motility assessment system (E and F; Song et al., 2019) of the herd over 66 d from March 8 to May 12, 2019. The output data include (A) milk yield (kg), (B) milk fat content (%), (C) milk protein content (%), (D) milk fat-to-protein ratio, (E) estimated ruminal contraction frequency (min^−1^), and (F) surface concavity of the left paralumbar fossa as the estimated rumen fill (mm^−2^). The negative, zero, and position values of the skewness indicate that the distribution is right-skewed, symmetrical, and left-skewed, respectively. A kurtosis of 3 indicates that the outliers are similar to that in a normal distribution. A kurtosis of <3 or >3 indicates light-tailed and heavy-tailed distributions, respectively. The farmer allowed all cows to graze on d 10 and replaced hay with corn silage from indoor feeding on d 25.

To investigate the association between a cow's rumen function and its milk production performance, we deployed the cross-correlation function “xcorr” in MATLAB (R2018b, MathWorks). A cross-correlation measures the similarity of 2 time-series data sets by calculating the time difference between the 2 sets having the best fit. Each time-series data set consisted of the herd-median value of a collected variable of all cows on each day. Upon time shifting the first data set (e.g., estimated rumen fill) forward and backward from 0 to 65 d, the cross-correlations to the second data sets (e.g., milk yield) were calculated. The highest absolute cross-correlation was considered the best fit of the 2 data sets. When this cross-correlation was significant (*P* < 0.05), its corresponding shifted days (i.e., peak lag time) represented the number of days elapsed until milk production (i.e., milk yield, fat content, and protein content) responded to a deviation in rumen function (i.e., estimated ruminal contraction frequency and rumen fill).

During the 66-d observation period, the 25th, 50th, and 75th percentiles of the estimated ruminal contraction frequency (Figure 1E) of the herd remained at approximately 3.0, 3.1, and 3.2 contractions per minute, respectively. These values were similar to the normal ruminal contraction frequency range (i.e., 1 to 3 contractions per minute), including primary and secondary contractions (Nagy, 2017). We expected the 3D vision system to capture both primary and secondary contractions because the focused body region of the 3D camera was the dorsal sac of the rumen, which contracts in both primary and secondary contractions. Moreover, the measured ruminal contraction frequencies were relatively high, which could be explained by cows being fed concentrate feed during video recordings. Additionally, the skewness of the distribution from the estimated ruminal contraction frequencies of all cows on each day remained close to 0 and the kurtosis was around 2, indicating a stable data set with relative symmetrical distribution and fewer outliers than a normal distribution (Figure 2E). Furthermore, because of the stability of the herd-level ruminal contraction frequency, we could not find any significant cross-correlation between the estimated ruminal contraction frequency and milk production. As the drastic deviations in ruminal contraction frequency are mainly associated with ruminal diseases or dysfunction, the stability of the measurements in this study indicated that most cows in the herd have no ruminal problems.

The herd median estimated rumen fill started to decrease from d 11 onward for 13 d when all cows were allowed to visit the pasture on d 10 (Figure 1F). On d 24, half of the cows had a concave paralumbar fossa surface, denoting low rumen fill. We speculated that the decrease in estimated rumen fill was associated with the onset of grazing, which was the only change in feeding practice during those days. At the start of grazing, some cows might have needed more time to adapt to fresh grass in their diet and to adjust their eating patterns, which could have resulted in low feed intake and low rumen fill. It is also possible that the ryegrass on the pasture was immature in early spring (i.e., March). Although the amount of indoor feeding remained, cows spending one-third of their time every day grazing immature grass could increase the rumen passage rate and result in low rumen fill. Additionally, the low rumen fill could be caused by insufficient NDF from the ryegrass-based pasture (Kendall et al., 2009). Although no pasture test was performed, we speculated that the NDF content could be less than 35%. From d 25 onward, the median of the paralumbar fossa surface started to change to convex (Figure 1F), and the skewness of the daily distribution changed from negative to 0 (Figure 2F). These slow changes indicated that cows started increasing their rumen fill over time, especially those with low rumen fill. The improvement in rumen fill could be because the grass was mature with more nutrients or because vulnerable cows were becoming used to digesting fresh grass.

The herd median milk yield decreased slightly over the 66 d from approximately 26 to 24 kg/d. Moreover, the decrease in milk yield in the 25th percentile was greater than the corresponding decreases in the 75th percentile and the median of the herd (Figure 1A). The distribution of the herd milk yield on each day had skewness decreased from positive to approximately −1 and stable kurtosis >2 (Figure 2A). These trends indicated that the decrease in herd-level milk yield was mainly caused by cows with relatively low milk yields, which were affected by grazing to a greater extent than the other cows. Additionally, the peak lag time was 14 d, with the highest absolute cross-correlation of 0.39 (*P* < 0.05) between the estimated rumen fill and milk yield over the 66 d. The highest cross-correlation was less than moderate (0.50), and a 14-d response time was long; hence, it was unlikely that the deviations in rumen fill had a direct or rapid effect on cows' milk yield.

The median of the milk fat content of the herd started to decrease from 4.7% on d 11 to 4.3% on d 13 (Figure 1B) and increased slowly to 4.6% on the last day. These deviations were mainly caused by the majority of the cows in the herd because the distribution of the milk fat content had a stable skewness around 0.5 and a kurtosis around 2 (Figure 2B). Additionally, the highest absolute cross-correlation between the estimated rumen fill and milk fat content over the 66 d was 0.58 (*P* < 0.05), with a peak lag time of 1 d. Because of this strong cross-correlation and short peak lag time, we speculated that the changes in feeding practice caused the lowered rumen fill, which led to deviations in the milk fat content. In particular, the milk fat depression was likely associated with immature ryegrass at the start of grazing in the early spring season, resulting in an NDF intake lower than that associated solely with indoor feeding (Kendall et al., 2009). Additionally, pasture grazing could cause an increase in PUFA (e.g., α-linolenic acid) in milk. This alteration in the composition of fatty acids in milk could have changed the milk color and caused biased fat content measurements. The milk protein content remained steady over the 66 d (Figure 1C) and had no significant cross-correlation with estimated rumen motility. The protein depression in milk likely starts slower than that of milk fat and, generally, only prolonged diet changes could cause milk protein depression (Leskinen et al., 2019). The milk fat-to-protein ratio followed the same pattern as the milk fat content (Figure 1D) because the milk protein content remained steady.

Among all 42 cows, one cow was identified as unhealthy and having abnormalities in awareness and ear temperature on d 52 based on our general health check. According to the farmer's on-farm management records, this cow was suspected of suffering from rumen dysfunction and received oral sodium bicarbonate powder from d 52 to 54. The changes in all measured variables of this cow were similar to the herd-median trends until d 41. From d 41 to 47, the estimated ruminal contraction frequency first decreased and then slightly increased (Figure 3E), whereas the estimated rumen fill first slightly increased and later markedly decreased (Figure 3F). It is likely that the cow had low rumen motility at first and then low rumen fill, indicating a deterioration in rumen function. The farmer noticed this cow on d 48 because of a decrease in milk yield (Figure 3A) but could not detect any clinical signs of disease. This record was consistent with our general health check performed on the same day (the cow had a health score of 6, denoting that it was healthy). On d 50, the cow exhibited a further decrease in milk yield as well as deviations in milk fat and protein contents. With no other supporting information available, the farmer suspected that the cow had mild mastitis and subsequently applied mint cream on the udder to prevent the cow from developing severe mastitis. On d 52, the veterinarian in training observed that the cow was dull and had cold ears. The associated health score was 4, denoting the likelihood of a health disorder. Additionally, the farmer registered watery manure from this cow and suspected it had rumen dysfunction. Subsequently, he offered 1 kg of additional hay, removed the concentrate supplements from the feed, and administered oral sodium bicarbonate to the cow for 3 consecutive days to buffer the pH in the rumen in an attempt to stimulate rumen motility. Also on d 52, both the estimated rumen fill and milk yield of this cow reached the lowest level. On d 54, the cow's rumen started to fill, whereas the estimated ruminal contraction frequency remained high. During and after treatment by the farmer, the milk fat content, protein content, and fat-to-protein ratio strongly increased and remained high, indicating that the cow did not fully recover. We suspected this cow of having rumen dysfunction, which was likely caused by changes in feeding practices: grazing on d 10 and hay replacement on d 25. Additionally, the cow could have had insufficient ability to overcome the corresponding challenges compared with others in the herd. The 3D vision system showed marked deviations in both the estimated ruminal contraction frequency and rumen fill from d 48, indicating a deterioration in rumen function. These deviations could be a sign for the farmer to detect this cow earlier. Nevertheless, with the currently available systems and sensors on the farm, the farmer could not perceive any sign of a ruminal disorder or perform targeted and effective ruminal dysfunction treatment.

**Figure 3:**
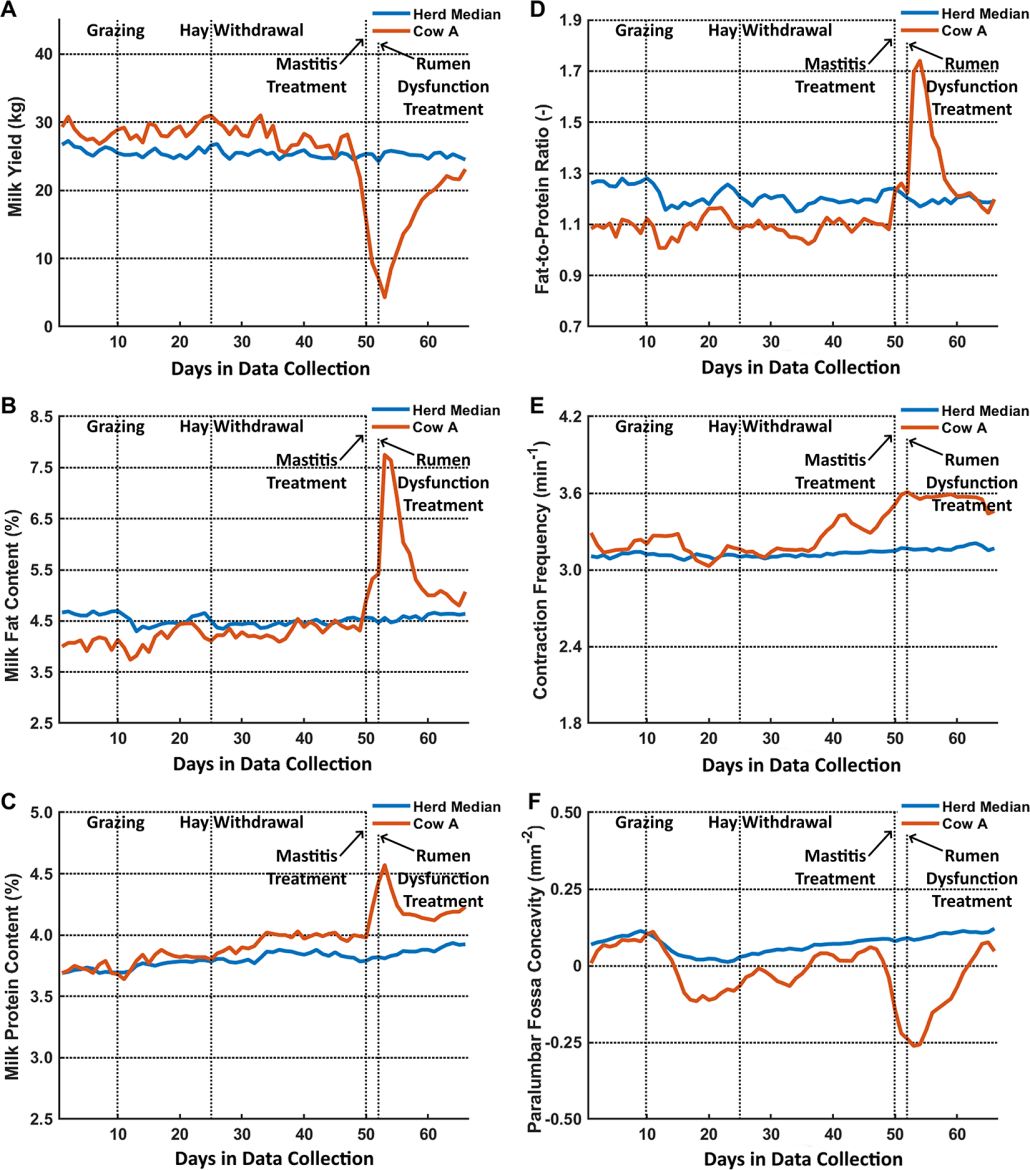
Plots of the outputs from the farm management software (A to D; T4C, Lely Industries N.V.) and 3-dimensional (3D) vision-based rumen motility assessment system (E and F; Song et al., 2019) of an unhealthy cow (cow A) over the 66 d from March 8 to May 12, 2019. The output data include (A) milk yield (kg), (B) milk fat content (%), (C) milk protein content (%), (D) milk fat-to-protein ratio, (E) estimated ruminal contraction frequency (min^−1^), and (F) surface concavity of the left paralumbar fossa as the estimated rumen fill (mm^−2^). In each plot, the cow was compared with the herd median (n = 42). The farmer allowed all cows to graze on d 10, replaced hay with corn silage from indoor feeding on d 25, suspected cow A of experiencing mild mastitis and applied mint cream to the udder on d 50, and suspected cow A of experiencing rumen dysfunction and administered oral sodium bicarbonate on d 52.

This study explored the associations between 3D vision-based rumen function assessment and changes in dairy cow feeding and milk production. The 3D vision system showed that half the cows in the herd had decreased rumen fill when they began grazing, which resulted in milk fat depression 1 d later. The decline in rumen fill could be caused by decreases in feed quality and the additional time that cows needed to adapt to changes in feeding practices. This speculation, however, has not been verified with feed quality assessments or individual cow feed intake measurements. Future studies need to focus on investigating the relationship between deviations in rumen motility and quantified adjustment in feeding practice (e.g., feed intake, pasture nutrient test, and nitrogen application rate), which is an essential step toward making the 3D vision system a valuable tool to assist farmers in feeding management. Moreover, the 3D vision system detected a cow with sudden decreases in estimated ruminal contraction frequency and rumen fill before detection by the farmer. It is likely that the cow had rumen dysfunction; however, it was only a single case without any clinical diagnosis to confirm. In future studies, we propose deploying rumen bolus systems with rumen motility, pH, and temperature measures to validate the 3D vision-based rumen motility assessment. Moreover, we suggest investigating more cases with ruminal dysfunction and ruminal diseases with clinical diagnosis as a reference to validate the 3D vision system in rumen function and health assessment. Because this study was performed on a limited number of cows and days, it would be worthwhile to further explore the feasibility of the 3D vision system as a farm management-supporting tool.
