# Erratum to “Automatic assessment of dairy cows' rumen function over time and links to feed changes and milk production” (JDS Commun. 3:126–131)

**DOI:** 10.3168/jdsc.2022-3-5-380

**Published:** 2022-09-12

**Authors:** X. Song, S. van Mourik, E.A.M. Bokkers, P.W.G. Groot Koerkamp, P.P.J. van der Tol

The animal ethics statement was missing from this paper. The following statement should have been included: **According to the Dutch legislation on animal experiments (Wet op de Dierproeven; WoD), this observational study is not considered an animal experiment. Therefore, no approval from the legal authorities was required**.
